# Sesquicarbonate of Ammonia in Lepra and Psoriasis

**Published:** 1852-07

**Authors:** 


From the London Lancet.

*Sesquicarbonate of Ammonia in Lepra and Psoriasis.*

M. Cazenave, so well known as a very successful dermatologist, has just published experiments tending to show that sesquicarbonate of ammonia may advantageously be used as a succedaneum of arsenical preparations, in lepra and psoriasis. The salt is mixed in the following proportions: Half a drachm of sesquicarbonate of ammonia; diaphoretic syrup, seven ounces; take from one to three tablespoonfuls per diem. The physiological effects are very slight, but in the space of about a week the scales begin to fall off; those that succeed are thinner, the patches which give them support gradually fall in, the redness fades after a longer or shorter time, and a lasting cure generally ensued. If diarrhœa, lassitude, cephalalgia, quick pulse, and rapid alternations of heat or cold, were to occur, as was the case with two or three patients, the remedy should be suspended.

